# Escalating post-disaster rescue missions through ad-hoc victim localization exploiting Wi-Fi networks

**DOI:** 10.1016/j.heliyon.2022.e09314

**Published:** 2022-04-29

**Authors:** Taslim Arefin Khan, Tarik Reza Toha, Saiful Islam Salim, Md Toki Tahmid, A.B.M. Alim Al Islam

**Affiliations:** Department of Computer Science and Engineering, Bangladesh University of Engineering and Technology, Dhaka, Bangladesh

**Keywords:** Search and rescue, Indoor localization, Prototype, WiFi network, IoT

## Abstract

The number of disasters, accidents, and casualties in disasters is increasing, however, technological advancement has yet to ripe benefits to emergency rescue operations. This contrast is even more prominent in the Global South. The consequences are a huge loss of wealth and resources, but more importantly, the loss of lives. Locating victims of disasters as quickly as possible while speeding up rescue operations can lessen these losses. Traditional approaches for effective victim localization and rescue often requires the establishment of additional infrastructure during the construction period. Which in the context of countries of the global south such as - Bangladesh, is not followed for most of the industrial and household constructions. In this paper, we conduct a study to better understand the challenges of victim localization in emergency rescue operations and to overcome them using “whatever” resources available at hand without needing prior infrastructure facilities and pre-calibration. We design and develop a solution for this purpose and deployed it in several emulated disaster-like scenarios. We analyze and discuss the results obtained from our experiments. Finally, we point out the design implications of an infrastructure-independent and extensive emergency rescue system.

## 1 Introduction

In the year 2016, Bangladesh witnessed 5,878 fire accidents claiming 1,609 lives and nine earthquake incidents claiming nine lives [3]. The number of death tolls in countries such as Bangladesh, is alarmingly high. What is more concerning is the frequency at which such disasters and accidents are happening, which too is alarmingly high. In the wake of such tragedies, preparedness for disaster and risk mitigation have been emphasized around the world. In the context of Global South where population is huge and the economy is growing, preparedness-only measure is not enough. Our focus has thus shifted to systems enabling emergency rescue operations.

Emergency rescue systems are crucial to post-disaster management. One of the most important tasks in emergency rescue work is locating victims of the disaster and rescue to save lives. Technological development in recent years have made it possible for rescue workers to use sophisticated machineries. However, locating victims in the wild is still a challenging problem. The concept of victim localization is closely related to indoor localization. However, indoor localization in the context of emergency scenarios is different and challenging. This is because disaster sites are generally unknown to the rescuers, structural collapses can cause debris and object-cluttered environment. Besides, fire hazards in many disaster-affected sites often lead to dense smoke and high temperature [23]. Withstanding such non-deterministic diversities makes localization in disaster-affected settings a challenging research problem. This problem demands exploration in the literature because of the intricacies and threats poised to the Global South.

Considering state-of-the-art indoor localization mechanisms and the challenges in emergency rescue scenarios in the Global South, two types of challenges stand out. First, the unpredictable nature of the emergency situations and what comes afterwards. For example, post-fire or earthquake incidents may not only cause danger to lives, it may also cause structural damage to the site. The second challenge is an extension of the previous. While designing a technology to withstand and aid any potential emergency situation is challenging, it is worth considering to what extent such technology can be made available and deployable at large to a dense population in the Global South. Bangladesh, for example, has approximately 163 million people to-date with 1,251.8 people per square kilometer area [4]. The challenge to us is the design of a technology that is pervasive and available to the mass. To address the first challenge, an ad hoc or infrastructure-independent mechanism can potentially come to aid in any unforeseeable consequences. For the second challenge, when we keep in mind the socio-economic conditions of Global South, a nation-wide policy and change-making to prepare and develop a drastically new mechanism for fast rescue and recovery is unlikely. Ideally, we would want a technology to be easy to use, deployable, and available to the mass without incurring huge setup cost.

In this paper, we take a step towards raising this problem for discussion and attention in the pervasive computing. We seek answers within existing technological ecosystem. Given the population boom and increase in both natural (e.g., earthquake) and accidental (e.g., fire) disasters, plus a shortage of rescue workers over the years, our journey through this problem was driven by “how might we” design approach. We contribute a design study of an emergency rescue system addressing the needs of Global South while we ask the following questions - “How might we save victims of a disaster without taking help from any pre-built or pre-established infrastructure? How might we take advantage of victim's smartphone to use as proxy to find victim's location?” The culmination of answering these two questions led us to design a system that can “save people using their own resources.”

We develop a prototype to use as a probe in this design study. Our contributions in this paper are -

1. informing the challenges and design of an emergency rescue system that is independent of pre-established infrastructure,
2. developing a prototype solution withstanding those challenges, and
3. conducting experiments in ten different testbeds emulating disaster-like scenarios to probe design challenges and demonstrate applicability of our prototype solution.

## 2 Background

This study is situated at the backdrop of on of the most tragic industrial accidents in Bangladesh. Structural collapse of Rana Plaza [5] caused the loss of more than a thousand lives. Rescue work at Rana Plaza stretched for 18 consecutive days.

We conducted an informal interview with a personnel from Bangladesh Fire Service and Civil Defense. There are 30 units of Fire Service stations in the capital Dhaka city. That is roughly one unit per 10 square kilometers area of Dhaka city. Given that the mega city is densely populated (even more than 1,251.8 people per square kilometer area nation-wide [4]), the Fire Service personnel pointed out that all the units are severely understaffed. Currently all substations are required to have 22 people in total. This requirement was framed back in 1992 and has not changed since then. According to their drill order in the Standing Operation Procedure [3], the first fire truck to arrive at a disaster site comes with 7 on-board personnels. Although subsequent fire trucks do follow based on necessity, this however points us to how many people engage in search and rescue missions.

In this study, although we take perspectives and design ideas by studying search and rescue missions in Bangladesh, our hope is that our design ideas will be equally applicable in the Global South.

## 3 Related work

We consider the bodies of indoor localization, localization in emergency scenarios, and support for emergency rescuers as areas of relevant research.

### 3.1 Indoor localization

Localization in indoor environment is a fundamental research problem. Research in this area developed based upon some fundamental basics - for example, signal strength, inertial or motion sensors, physical information of the radio channel, are few well known approaches. Studies such as [13] proposed deploying external infrastructures to monitor the signal variation in the survey site. Two key components for infrastructure monitoring are Wi-Fi monitors (sniffers) and signal map reconstruction [13]. However, infrastructure-based monitoring brings extra deployment cost, which may not be suitable for large survey site. Some non-infrastructure based models have also been proposed. Here, the offline training and online localization complexity is relatively high. Crowdsourcing approaches are great alternatives to avoid such problems. Contrary to these approaches where a calibration phase is inevitable, some other work [17] emerged that leverage smartphone's inertial sensors with or without crowd-powered techniques to localize in indoor settings. In recent times, Received Signal Strength (RSS) based wifi indoor localization has gained much interest. While most of the works tend to minimize the localization error, [26] examines the diversity of Wi-Fi signal distributions and the measurement error associated with RSS values. A hybrid hypothesis test leveraging the idea of Asymptotic Relative Efficiency is designed by considering the various access points of the indoor environment to minimize the errors resulting from varied wifi signal distribution and complicated surroundings. In [10], a system is developed for accurate indoor localization of people visiting a museum or any other cultural institution. The performance results obtained from measurements show an achievable position estimate accuracy below 1 m. However, it was assumed that visitors are equipped with a Bluetooth Low Energy (BLE) device provided while entering. In recent years, cost effective accurate indoor localization using machine learning has gained significant interest. In [19], machine learning based indoor localization (MLBIL) techniques is proposed for cost effective accurate indoor localization. Analyzed features for the technique is categorized into: RSS learning, Non RSS learning, and Multiple feature learning. A recent work [2] proposes the use of geomagnetic field patterns called MP (Magnetic Pattern) with CNN (Convolutional Neural Networks) to perform indoor localization.

### 3.2 Localization in emergency scenarios

Locating victims in disaster affected environment received much less attention than indoor localization in general. When some of the infrastructures are down on account of a disaster, the effects it have on localization accuracy has been modeled in [20]. They used the Pedestrian Dead Reckoning and Wi-Fi RSSI fingerprinting models to simulate indoor localization. A hybrid algorithm has been proposed to provide location information of victims to rescue workers using time-of-arrival and received power of GSM network [21]. They simulated and showed the trade-offs between location error and path-loss exponent. A similar work with real deployment has been presented in [27]. In this work, a local GSM base station was deployed and directional antennas were used to locate victim's mobile phone. A camera based victim localization model is proposed in [25], where the localization error is not specified explicitly. In [11] a system design is proposed that uses the smartphone Wi-Fi AP for on-site location identification and response. Here the minimum localization error was found 4.137 m. Wi-Fi fingerprint data is used and analyzed to track the victim's position. In [15], an android app is used to detect several victims trapped under the WLAN network of a building by using Euclidean Distance (ED) algorithm. In recent times, building information modeling (BIM) has gain significant consideration in industry as a central repository of building information. In [9] a BIM-based Indoor Location (BIMIL) protocol is designed for automated data extraction and transformation of BIM emergency-related data for public safety purposes. This approach can help to localize crucial portions of a disastrous construction site with indoor positioning data to support emergency responses. Availability of an up-to-date layout of a building is crucial for faster rescue management. After localization, access to an optimized path to the victim plays a significant role for emergency support. Often due to structural and interior changes inside a building it is difficult to maintain a synchronic layout. One probable approach is to reconstruct the 3D models of the building after any renovation in an automatic approach. In [16], a complete workflow is introduced that generate 3D models from point clouds of a building to support sophisticated path planning for disaster management. However, this study does not anyhow facilitate localizing the victims.

### 3.3 Support for emergency rescuers

The study in [7] proposed a WSN based support for emergency responders or rescuers. It proposed a joint routing and localizing algorithm based on pre-deployed Wi-Fi network. The study in [24] proposed an inertial sensor based technique to localize first-responders in disaster scenario. For inertial sensors such as, accelerometer, gyroscope, and magnetometer to work, a centralized system needs to know the starting position of the first-responder. Moreover, continuous sensing is required.

In our study, we primarily focus on locating victims in disaster affected areas. We built a prototype to probe design of an emergency rescue system that could accept challenges of working in infrastructure independent situations. In our case, an ad hoc calibration step is necessary. We will show in later sections how our proposed design works. We also introduce a probing step where we concentrate on a search zone. The probability of finding victims in that search zone thus increases. Like most other work, we also take advantage of Wi-Fi RSSI since smartphones are ubiquitous and widely available. We name our prototype as VLoc (**V**ictim **Loc**alization). In Table 1 we show comparison of VLoc with other studies. VLoc is different from other studies pertinent to localization in disaster-affected settings in that, it does not require additional infrastructure, and unlike other studies, VLoc narrows down search zone by probing for victims and identifying a search zone of interest. We report in Table 1 the best case location error across several trial runs of VLoc in order to set a comparison with other mechanisms reported in the literature.

**Table 1:** Comparison of VLoc with existing studies.

Approach	Focus	Additional Infrastructure	Prior Anchor Nodes	Pre-calibration	Search Zone Probing	Location Error	System Used
VLoc	Disaster - indoor	No	No	Ad hoc and quick	Yes	0.82 m	Wi-Fi and ad hoc
Tassetto et al. [21]	Disaster - outdoor	No	No	Not needed	No	Unspecified	GSM
Zorn et al. [27]	Disaster - outdoor	Yes	No	Pre-planned	No	0.10 - 1.00 m	GSM
Vemula et al. [22]	Disaster - indoor/outdoor	Yes	No	Pre-planned	No	3.65 m	RFID
Zhang et al. [25]	Disaster - indoor	Yes	Yes	Pre-planned	No	Unspecified	Camera
Ko et al. [11]	Disaster - indoor	Yes	No	Pre-planned	No	4.137 m	Wi-Fi
Mutiawani et al. [15]	Disaster - indoor	Yes	No	Pre-planned	No	Unspecified	WLAN
Giuliano et al. [10]	Security - indoor	No	No	Pre-planned	No	1 m	Bluetooth

## 4 VLoc: a design study

The goal of this study is to inform the design of a victim localization mechanism that is - (1) not dependent on any pre-installed infrastructure, and (2) exploits pervasive devices and networks, such as smartphones and Wi-Fi. We achieve this by building a prototype as a design probe to understand what could work well and not.

**Figure 1:**
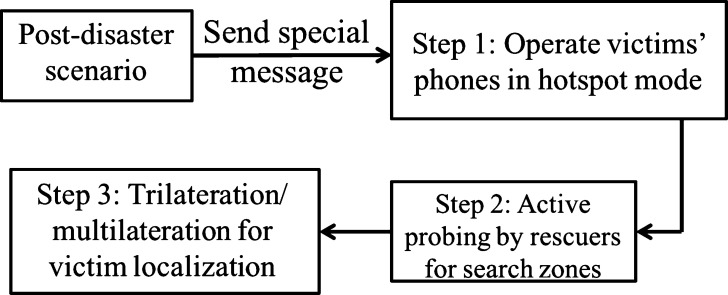
Functional overview of VLoc.

Fig. 1 shows the functional overview of VLoc. During a post-disaster situation, a text message (SMS) is sent to victims' phone number from a number registered for emergency purposes (e.g., 999 in Bangladesh). An app installed in the victim's smartphone intercepts this text message and switches on the Wi-Fi hotspot. Operating victims' phones in this mode is crucial for locating one or more victims' with their approximate locations in the disaster site. Once an approximate location is identified, rescuers leverage trilateration or multilateration techniques on the Received Signal Strength Indicator (RSSI) from the Wi-Fi signal of victims' phones. VLoc estimates the current location of a victim from three or four reference points in trilateration or multilateration. The reference points are the rescuers and VLoc provides the distance in meters to the victim from all rescuers.

### 4.1 Apparatus used in our design and development

**Figure 2:**
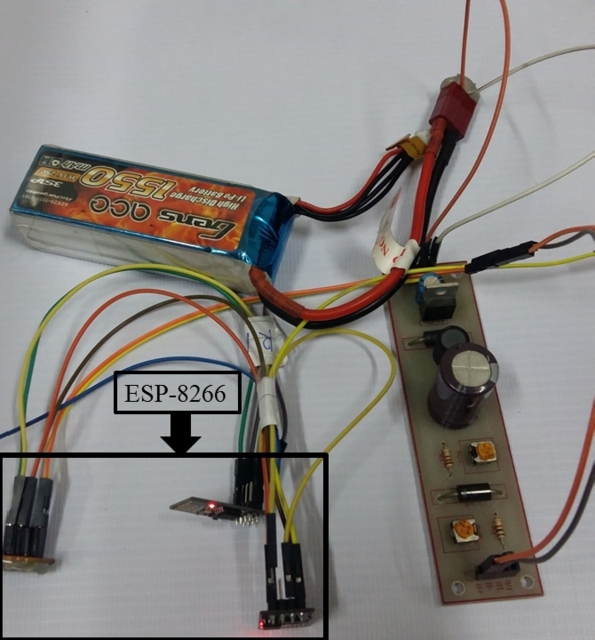
Custom-built ESP 8266 Wi-Fi module.

We use a custom-built ESP 8266 Wi-Fi module to change Wi-Fi settings. ESP 8266 is easy to customize and programmable. Fig. 2 shows the ESP 8266 Wi-Fi module connected to a Lithium Polymer battery. We use this module to selectively work either as a Wi-Fi Access Point (AP) or as a client. We use several pieces of this module because of low cost, ease of customization, and deployment flexibility. We use a Samsung A5 smartphone device updated to Android version 6.0.1, equipped with Quad-core 1.2 GHz Cortex-A53 CPU and 2 GB RAM. This smartphone is also used selectively as AP or client. Finally, we use an Acer Aspire 5745 laptop for data collection. We connected one ESP 8266 module via USB to this laptop to capture RSSI data from other Wi-Fi APs. We maintain consistency in data collection by assigning each module to a designated purpose. For example, all custom ESP 8266 modules are pre-assigned for roles as victims or rescuers.

Note that, in our proposed solution, the ESP 8266 module (or similar custom solution) will solely be used by the rescuers. Here, the ESP module (or similar custom solution) will be used by the rescuers for integration with a hotspot network. The hotspot network will be created by the mobile phone of the victim upon reception of a message through our designated app.

In our experimentation, to better tune up Wi-Fi settings and to perform necessary measurements (such as RSSI), we use ESP modules both in places of rescuers and victims in some cases. Here, in the case of victims, our used ESP modules actually mimic the Wi-Fi modules of the mobile phones of the victims. Thus, even though some of the ESP modules in our experiments are used in place of some of the victims for the purposes of ease of changing settings and having measurements, they will not be used to victim sides in real cases in our proposed solution.

### 4.2 How VLoc works

In the following, we explain each steps mentioned in Fig. 1.

#### 4.2.1 Step 1: Operating in hotspot mode

**Figure 3:**
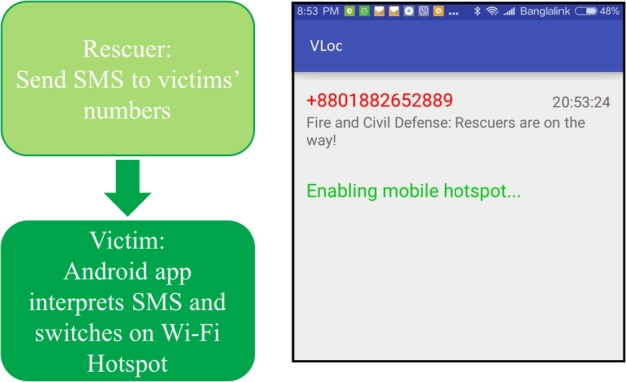
Switching to Wi-Fi hotspot mode in victim's phone by sending an SMS.

The first step is to operate victims' smartphones in hotspot mode. We develop a prototype app in Android as shown in Fig. 3. Here, we assume that rescue workers have phone numbers of all the victims trapped inside the disaster site. The aim is to operate victims' phones in hotspot mode. This will allow victims' smartphones to work as Wi-Fi Access Points (APs). Wi-Fi APs will help VLoc in two subsequent steps - probing for victims and locating victims. Note that, continuously running phones in hotspot mode could drain battery life, we use SMS as an external trigger to switch on hotspot mode when necessary.

#### 4.2.2 Step 2: Probing for victims and identifying a search zone

The purposes of probing for victims are to identify a region to search for trapped victims and to escalate rescue process by focusing on those regions only.

**Figure 4:**
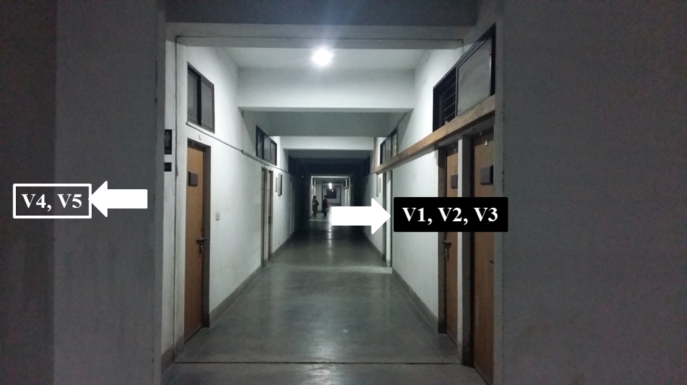
Positioning five victims in two groups in a laboratory to demonstrate how probing for victims work.

Fig. 4 shows a laboratory setup where we demonstrate how probing for victims work in VLoc. Our idea is simple. Since victims' phones are operating as Wi-Fi APs, it is possible to measure RSSI from these APs using another Wi-Fi device. This is done by one or more emergency rescuers. In Fig. 4, V1-V5 are the labels of five victims. For the sake of a simple demonstration, we split this group of five into two, putting three victim APs in one side of the corridor and the rest on the other side.

**Figure 5:**
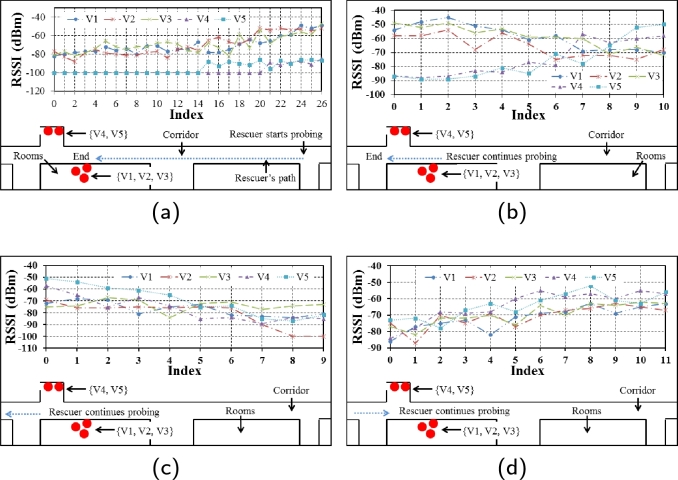
Real time RSSI data captured from nearby victim APs by a Wi-Fi client device carried by a rescuer. (a) Rescuer walks towards the other end of the corridor and discovers two sets of RSSI data getting stronger, (b) Rescuer continues in the same path while RSSI of V4, V5 strengthens and V1, V2, V3 weakens, (c) Walking further down the corridor shows both sets weaken, (d) Returning back through the same path shows both sets getting stronger again.

In Fig. 5a-d, we show a simple demonstration of how we expect a working system to function. Here, the X axis shows the time index. This time index increases in proportion to the trail followed by the rescuer (blue dotted line). Probing for victims works under the assumption that victims' phones are working in hotspot mode. We also assume that victim APs have a known SSID, which VLoc is able to parse in the rescuer's device. In real life scenario, a rescuer may not know which way to start searching for victims. Therefore, movements of rescuers will be random. We take this random movement into account and develop a human-in-the-loop system to determine which areas to consider as potential search zones. Fig. 5a-d shows different movements of a rescuer and how the five signals from five APs look like on his device. Consider Fig. 5b where the rescuer seems to pass by the place where V1-3 are located and getting closer to where V4-5 are located. Consequently, the RSSI of V1-3 becomes weaker whereas V4-5 becomes stronger. All the signals become weaker in Fig. 5c as the rescuer moves away from them. When the events from Fig. 5b and c are taken into account together, it leaves us a hint that the rescuer is gradually moving away from an area potentially hosting five victims.

**Figure 6:**
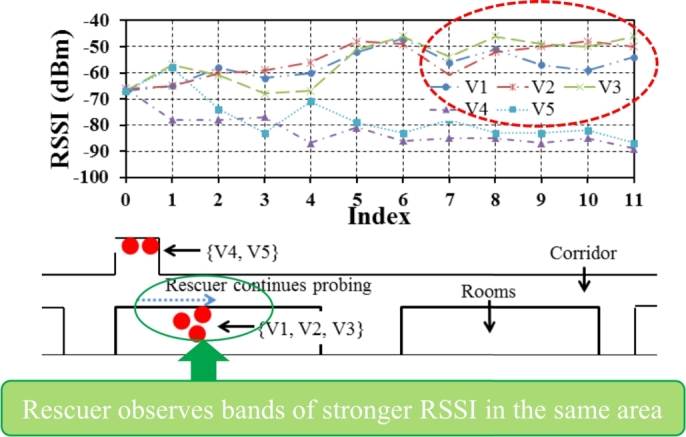
Real-time RSSI data shows band of strong signals in the same location he walked by before.

How does the rescuer know when to stop and be certain that there are victims nearby? We take a trial and error approach for this purpose. Rescuer may walk in front of the anticipated zone a few more times to see the same pattern of signals. In Fig. 6, the rescuer comes back in anticipation and observes similar bands of strong RSSI in the same area. In this way, the rescuer can identify a potential search zone and look for victims in that zone only. In the next section, we explain the third step, how to locate a victim in that zone.

## 5 Locating victims in search zone

The final step in VLoc is locating victims within a potential search zone. We take into account feasibility of existing indoor localization mechanisms under emergency situations. We also take into account the nature of indoor environments and assess the feasibility of a system where we can exploit an ad hoc setup, such as smartphones' Wi-Fi signal in hotspot mode.

### 5.1 Path loss models

The behavior of radio signals (RF) in any indoor environment is lossy [18]. Ceiling, floor, walls, furniture, people, etc., affect electro-magnetic wave propagation. Based on the nature of the environment, different elements have been modeled individually and used where suited. Several models have been proposed in [18]. These models consider signal attenuations as path loss. Path loss is defined as the difference (in dB) between transmitted power and received power.

### 5.2 Log-distance path loss model

The log-distance path loss model has been considered by several other research studies for indoor localization [6]. We also consider this model to estimate unknown distances from RSSI values. Compared to other models, log-distance path loss model is relatively simple. This model formulates path loss as follows:

(1) $$PL(d)=PL(d_{0})+10nlog\left(\frac{d}{d_{0}}\right)+X_{\sigma }$$

In this equation, PL(d) is the path loss at distance *d*. Here, the path loss is defined as the difference (in dBm) between transmitter (T_*x*_) and receiver (R_*x*_). Similarly, PL(d_0_) is the path loss at distance d_0_, where d=01 m. Moreover, the path loss exponent n=4 is common in indoor space [8]. X_*σ*_ represents a normal random variable in dB with a standard deviation of *σ* dB. In indoor spaces with no path loss, the value of *σ* is zero. In other cases, it takes the form of a Gaussian distribution with standard deviation *σ* dB. In our study, we calculate *σ* by performing an initial calibration. There are slight deviations for the value of *σ* in each of the testbeds where we conducted our experiments. For example, in normal indoor office environments, the value is approximately 7.85. This value is very close to the reported value in [8]. Given all the values, we are interested for the unknown *d* in this equation.

### 5.3 Approaches to localization

We explore trilateration and multilateration [14] techniques for estimating the location of a victim using RSSI. We use the Log-distance path loss model to estimate the distances between the victim and a reference point.

**Figure 7:**
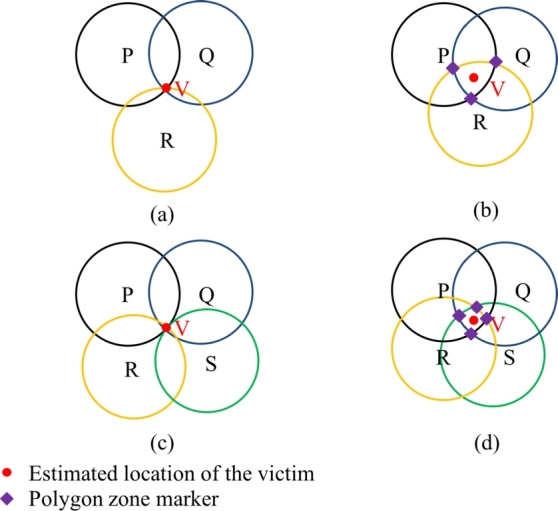
An illustration of four possible cases of victim localization. (a) Trivial case of trilateration - three circles coincide at a point, (b) Non-trivial case of trilateration where the overlapping region is bounded by three vertices, (c) Trivial case of multilateration where four circles coincide at a point, (d) Non-trivial case of multilateration where the overlapping region is bounded by four vertices.

Here, the reference points are the rescuers. When three rescuers act as references, we call it trilateration. When four rescuers act as references, we call it multilateration. Each reference points estimate a distance of the victim from its position, which gives a radius (or circle) around its position. When all the circles coincide at one point, we get the estimated location of the victim (Fig. 7a and 7c). It is also possible (and perhaps more likely) that the circles may overlap each other without coinciding at a particular point. In that case, the maximally overlapping zone is taken into account and the centroid of that region gives the estimated location of the victim (Fig. 7b and 7d).

### 5.4 Demonstrating VLoc

**Figure 8:**
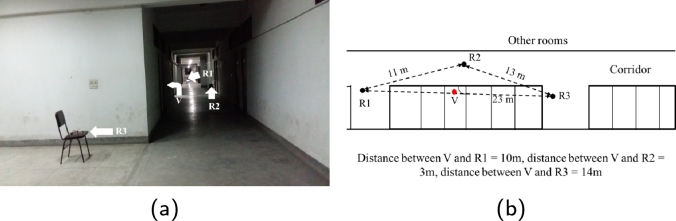
(a) A testbed setup with three rescuer and one victim device, (b) top-view of the testbed in (a).

**Figure 9:**
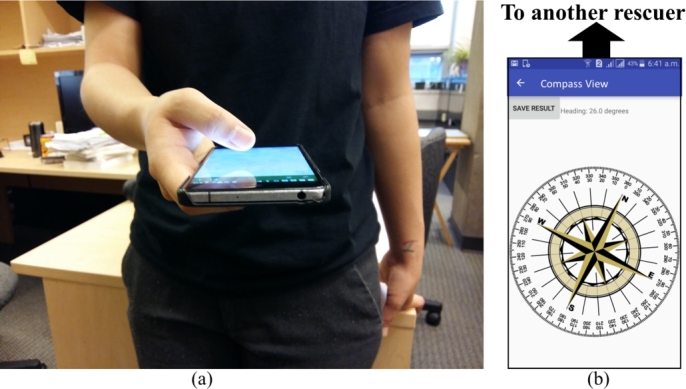
(a) A rescuer holds her mobile phone similar to resting a phone on a table-top on its back while pointing it to another rescuer, (b) Android app with compass showing the direction at which another rescuer is standing.

We demonstrate how VLoc works in a real scenario using our prototype. To demonstrate trilateration, we position three rescuers (R1, R2, and R3) around a potential location as shown in Fig. 8. While standing in their positions, rescuers will send the following data to a server - estimated distances to other rescuers, RSSI value from victim's phone, and cardinal direction (angle in degrees) to which other rescuers are positioned relative to himself. The last item is measured by a compass as shown in Fig. 9.

**Figure 10:**
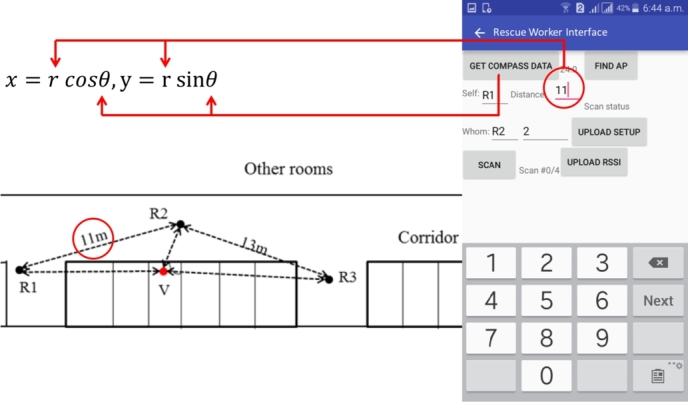
Rescuer R1 is logging distance and cardinal direction to R2.

How are these data captured and sent to the server in real-time? In Fig. 10 we show the screen of our prototype that helps rescuers to capture data and send to the server. Here, R1 is logging this data and he inputs an estimated distance of 11 meters from R2. Our informal interviews with local fire station officer revealed that firefighters are good at distance estimation based on their field experiences. There are existing smartphone apps that uses computer vision to measure distance from self to an object. This is beyond the scope of this research, we do not explore it further. As shown in Fig. 9, rescuer measures the cardinal direction of R2 with respect to himself. Besides, he also keeps recording RSSI from the victim. All of these data are then sent to the server.

**Figure 11:**
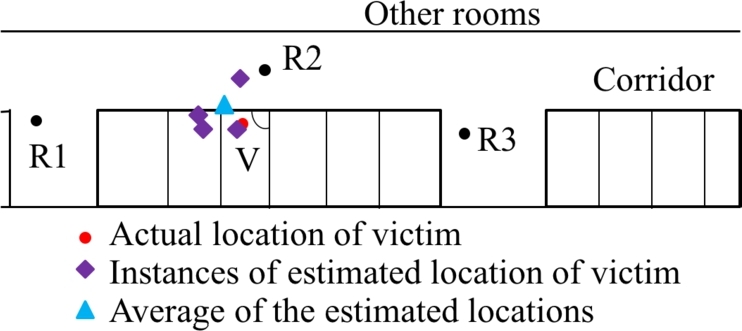
Location estimation using VLoc and the localization error.

What calculations are done in the server? The primary calculation that we are interested in is determining the distance of the victim from the rescuers. Since we have RSSI measured from the victim by all the rescuers, we can use Log-distance path loss model to estimate the distance. Referring back to Equation (1), we see that, for three rescuers, we can have a total of 27 instances (the mean *μ*, *μ*+*σ*, and *μ*-*σ*) that will provide estimated distances from the rescuers. However, not all of these instances have overlapping regions. We are only interested in those instances where an intersecting point or a common overlapping region is available. In our experiment, we have only four instances where we have valid overlapping regions. Fig. 11 shows the four instances.

How are these data points calculated? Two data points - approximate distance of self to another rescuer and cardinal direction of that rescuer from self are crucial in measuring a cartesian coordinate (*x*, *y*) from the polar coordinates. When we have (*x*, *y*) position of all the rescuers, it becomes easy to pin the estimated location in terms of (*x*, *y*). Note that, in our experiment, we always considered the position of rescuer 1 (R1) as the reference or (0,0). Positions of other rescuers (R2, R3, or R4 in multilateration) are measured with respect to this position. Thus, we now know how the estimated location in all instances are calculated. However, we cannot have multiple instances as a solution. Therefore, we calculate the average of all the instances. Now, we know the position ($x_{v}$, $y_{v}$) of this estimated location in two-dimensional space. VLoc also calculates the distances of this location from each rescuer's position. Therefore, for any rescuer, we know the distance to the victim $r_{v}$ and the position ($x_{v}$, $y_{v}$). Converting this coordinate to polar coordinate gives us the angle ($\theta_{v}$). The distance and the angle can guide the rescuers to the victim's estimated location. In this particular case, locating victims took approximately 6.9 minutes. We report this as response time in the next section.

### 5.5 Validating location accuracy and feasibility of VLoc

We perform several experiments to test location accuracy and feasibility of VLoc in disaster-alike situations. We took into account three key environmental features while setting up testbeds to perform experiments. First, we characterize some environment as non-line-of-sight or NLOS to radio propagation where the nature of RF is lossy. Second, we setup testbeds to incorporate line-of-sight or LOS characteristics. Finally, we also setup testbeds to fuse both NLOS and LOS characteristics. Consequently, we conduct our experiments in 10 different testbeds which we categorize into four groups.

1. Normal office environment (four testbeds, average area 33 $m^{2}$) - characterized by open or closed wooden doors, surrounded by concrete walls, glass windows with steel frames,
2. Disaster-like scenario with undamaged structures (two testbeds, average area 29 $m^{2}$) - characterized by closed doors and windows, highly congested and unorganized office materials,
3. Disaster-like scenario with fire (one testbed, area 70 $m^{2}$) - characterized by four large gas stoves, open doors and windows, enclosed by concrete walls, and
4. Disaster-like scenario with damaged or collapsed structures (three testbeds, average area 44 $m^{2}$) - characterized by typical outdoor space with under-construction materials (bricks, bamboos, steel and aluminum frames of various kinds, devoid of nearby human and other RF devices).

**Figure 12:**
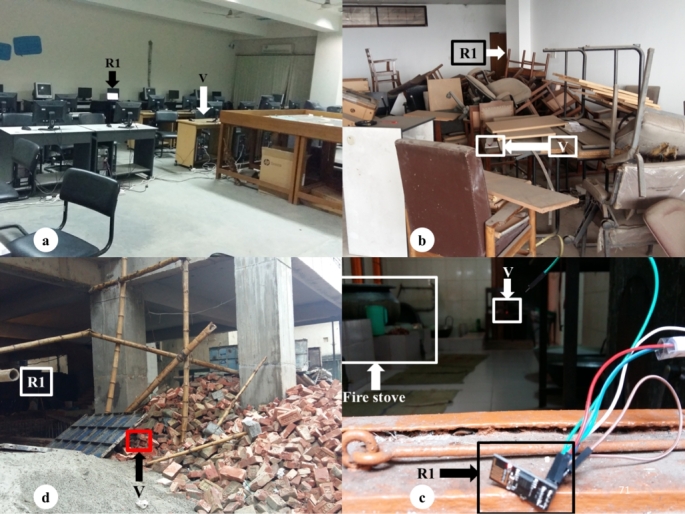
Testbeds for experiments. Clockwise from top-left: (a) normal office environment, (b) disaster-like scenario with undamaged building structures, (c) disaster-like scenario with fire, and (d) disaster-like scenario with damaged or collapsed structure.

Fig. 12 shows examples of one testbed from each of the four categories. In the next section, we summarize the results obtained from performing experiments in these 10 testbeds.

## 6 Results

In this section, we report average values and standard deviations (in brackets) of localization errors (L.E.) in meter and response times (R.T.) in minutes for all testbeds. Our reported values are average of three iterations of our experimentation. Note that, the response times are simple summation of times required in all steps conducted while locating victims in a search zone. Rescuers' data collection were done in a round-robin fashion, hence the ideal response times will be less when data collection is done simultaneously. We also tested the effect of co-located victims on localization error by increasing the number of victims from 1 to 4 in all experiments.

### 6.1 Category 1: Normal office environment

Table 2, Table 3, Table 4, Table 5 show the results of all four testbeds under normal office environment (e.g., Fig. 12a). Only Testbed 2 and 3 had LOS RF propagation. Other testbeds had NLOS RF propagation. The areas of these testbeds respectively are 40 $m^{2}$, 35 $m^{2}$, 22 $m^{2}$, and 36 $m^{2}$.

**Table 2:** Results of experiments conducted in Testbed 1 (area = 40*m*^2^).

# of Victims	Trilateration		Multilateration	
	L.E.	R.T.	L.E.	R.T.
1	2.2 (0.26)	6.9 (0.3)	2.30 (0.12)	9.5 (0.3)
2	2.09 (0.21)	6.7 (0.2)	2.01 (0.11)	9.1 (0.3)
3	1.24 (0.19)	6.8 (0.2)	2.45 (0.18)	9.4 (0.2)
4	1.28 (0.19)	6.3 (0.2)	2.52 (0.23)	9.5 (0.2)

**Table 3:** Results of experiments conducted in Testbed 2 (area = 35*m*^2^).

# of Victims	Trilateration		Multilateration	
	L.E.	R.T.	L.E.	R.T.
1	0.82 (0.12)	6.7 (0.2)	0.83 (0.07)	9.1 (0.4)
2	1.2 (0.2)	6.3 (0.1)	0.83 (0.09)	9.6 (0.5)
3	1.82 (0.14)	6.8 (0.2)	0.78 (0.11)	9.4 (0.3)
4	1.24 (0.27)	7.0 (0.4)	1.31 (0.41)	9.4 (0.4)

**Table 4:** Results of experiments conducted in Testbed 3 (area = 22*m*^2^).

# of Victims	Trilateration		Multilateration	
	L.E.	R.T.	L.E.	R.T.
1	1.33 (0.15)	6.3 (0.2)	1.83 (0.18)	8.8 (0.3)
2	1.48 (0.19)	6.4 (0.3)	1.04 (0.08)	9.3 (0.3)
3	1.52 (0.27)	6.7 (0.3)	1.28 (0.09)	9.2 (0.7)
4	1.48 (0.33)	6.3 (0.3)	1.16 (0.16)	9.0 (0.1)

**Table 5:** Results of experiments conducted in Testbed 4 (area = 36*m*^2^).

# of Victims	Trilateration		Multilateration	
	L.E.	R.T.	L.E.	R.T.
1	1.29 (0.02)	6.1 (0.4)	1.13 (0.11)	8.7 (0.2)
2	1.15 (0.07)	6.3 (0.3)	0.99 (0.03)	9.1 (0.3)
3	1.13 (0.11)	6.3 (0.4)	0.97 (0.10)	9.4 (0.5)
4	1.09 (0.04)	6.5 (0.2)	0.91 (0.06)	9.4 (0.2)

### 6.2 Category 2: Disaster-like scenario with undamaged structures

We setup two testbeds in an old warehouse where furnitures and electronic machineries are dumped haphazardly (Fig. 12b). These two testbeds are setup to emulate NLOS RF propagation. Table 6, Table 7 present results from the experiments conducted in these two testbeds. The areas of Testbed 5 and 6 are respectively 30 $m^{2}$ and 28 $m^{2}$.

**Table 6:** Results of experiments conducted in Testbed 5 (area = 30*m*^2^).

# of Victims	Trilateration		Multilateration	
	L.E.	R.T.	L.E.	R.T.
1	2.23 (0.18)	6.9 (0.3)	1.29 (0.37)	9.4 (0.3)
2	1.94 (0.08)	6.7 (0.5)	2.04 (0.81)	9.4 (0.7)
3	1.83 (0.49)	6.9 (0.2)	2.17 (1.05)	9.8 (0.4)
4	2.32 (0.36)	7.1 (0.3)	2.11 (1.19)	10.2 (0.3)

**Table 7:** Results of experiments conducted in Testbed 6 (area = 28*m*^2^).

# of Victims	Trilateration		Multilateration	
	L.E.	R.T.	L.E.	R.T.
1	2.14 (0.88)	6.7 (0.3)	0.54 (0.97)	9.3 (0.4)
2	2.21 (0.51)	6.9 (0.1)	1.47 (0.44)	9.7 (0.3)
3	2.36 (0.74)	6.4 (0.0)	1.31 (0.7)	9.9 (0.7)
4	2.26 (0.6)	6.1 (0.4)	1.54 (1.1)	9.5 (0.5)

### 6.3 Category 3: Disaster-like scenario with fire

The idea here is to emulate fire situation in laboratory conditions. Fig. 12c shows a kitchen space with four large gas stoves where we conduct our experiments. In this testbed (area 70 $m^{2}$), we conduct experiments under stove on and off conditions. However, we did not find any statistically significant interaction effect between the two conditions. Owing to limited space here, we only report ‘stove on’ condition in Table 8.

**Table 8:** Results of experiments conducted in Testbed 7 (area = 70*m*^2^).

# of Victims	Trilateration		Multilateration	
	L.E.	R.T.	L.E.	R.T.
1	3.43 (0.56)	7.1 (0.2)	3.29 (0.62)	10.3 (0.4)
2	3.58 (0.89)	7.5 (0.4)	3.95 (1.07)	9.8 (0.3)
3	3.52 (1.07)	6.9 (0.3)	3.63 (0.71)	9.4 (0.4)
4	3.63 (1.33)	7.2 (0.3)	3.49 (0.14)	10.1 (0.2)

### 6.4 Category 4: Disaster-like scenario with collapsed structures

We extend our experiments to emulate localization under collapsed structures. Fig. 12d shows one of the three under-construction buildings emulating collapsed structures. We show results from these experiments in Table 9, Table 10, Table 11. The areas of Testbed 8, 9, and 10 are respectively 63 $m^{2}$, 40 $m^{2}$, and 30 $m^{2}$.

**Table 9:** Results of experiments conducted in Testbed 8 (area = 63*m*^2^).

# of Victims	Trilateration		Multilateration	
	L.E.	R.T.	L.E.	R.T.
1	1.03 (0.28)	6.1 (0.8)	0.93 (0.12)	8.2 (0.4)
2	0.95 (0.11)	6.3 (0.2)	0.92 (0.07)	8.4 (0.3)
3	1.26 (0.44)	6.5 (0.3)	0.97 (0.24)	8.1 (0.4)
4	1.25 (0.69)	6.2 (0.3)	1.13 (0.33)	8.3 (0.5)

**Table 10:** Results of experiments conducted in Testbed 9 (area = 40*m*^2^).

# of Victims	Trilateration		Multilateration	
	L.E.	R.T.	L.E.	R.T.
1	2.72 (1.22)	5.8 (0.4)	2.46 (0.22)	8.5 (0.1)
2	2.87 (1.00)	6.2 (0.0)	2.59 (0.19)	8.2 (0.1)
3	2.62 (0.84)	6.0 (0.1)	2.67 (0.09)	8.2 (0.1)
4	2.57 (0.98)	5.9 (0.6)	2.71 (0.13)	8.1 (0.2)

**Table 11:** Results of experiments conducted in Testbed 10 (area = 30*m*^2^).

# of Victims	Trilateration		Multilateration	
	L.E.	R.T.	L.E.	R.T.
1	1.44 (0.32)	6.1 (0.4)	1.24 (0.12)	7.5 (0.4)
2	1.47 (0.05)	5.9 (0.3)	1.31 (0.09)	8.1 (0.0)
3	1.45 (0.70)	6.3 (0.2)	1.31 (0.22)	7.9 (0.2)
4	1.66 (0.14)	6.5 (0.3)	1.44 (0.26)	7.9 (0.3)

## 7 Discussion

In this section, we discuss about the results presented in the previous section, implications to design, and some avenues for further improvement of our work.

### 7.1 Energy efficiency and system resource utilization

Design of an emergency rescue and support system should first and foremost consider about energy efficiency and utilizing minimum system resources. Typical smartphones have a 3.8 V 2200-4000 mAh battery. Although Wi-Fi transmission power varies from vendor to vendor in smartphones, typically the average power consumption is 100 mW for 2 dBm transmission [12]. This only accounts for the Wi-Fi chipset and does not account for other overheads.

We analysed the resource utilization in VLoc in a smartphone. Table 12 shows CPU and RAM usage at different steps of VLoc. Here, the first three rows are pertinent to rescuer's device and the last row is pertinent to victim's device. The demand for computational resources is very low at any stage in VLoc. Moreover, RAM usage demand is also low considering the fact that most modern smartphones are now equipped with at least 1 GB or more memory.

**Table 12:** CPU and RAM usage at different steps of *VLoc*.

	CPU (%)	Memory (MB)
Calibration	2.24 (0.87)	12.16 (3.2)
Probing	2.3 (0.12)	14.53 (4.1)
Localization	1.87 (0.3)	12.94 (2.44)
WiFi hotspot	0.10 (0.008)	1.73 (0.49)

### 7.2 Variable transmit power

**Figure 13:**
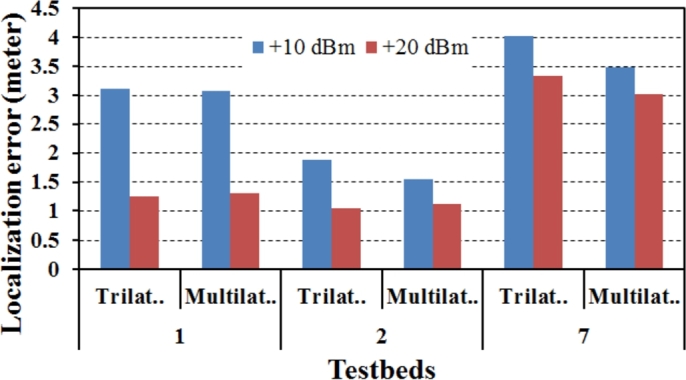
Localization errors at different power levels.

Different smartphones operate at varying Wi-Fi transmit power levels because of different chipset vendors. In case of smartphones, software defined programs can increase this power to 20 dBm. We designed our prototype in a way to accommodate different power levels. All the reported results in the previous section are, however, calculated at 15 dBm transmission power. Nonetheless, to analyse sensitivity to different power levels, we tested VLoc at 10 dBm and 20 dBm in Testbeds 1, 2, and 7. From Fig. 13 we see that at 20 dBm we have lesser localization errors. However, higher power comes at the expense of more battery consumption. Study suggests that per packet average transmission power of typical smartphones is around 2 dBm. The design of such system should opportunistically take advantage of high transmission power to accelerate rescue process by minimally impacting battery life.

### 7.3 How does arbitrary rescuer position impact localization?

**Figure 14:**
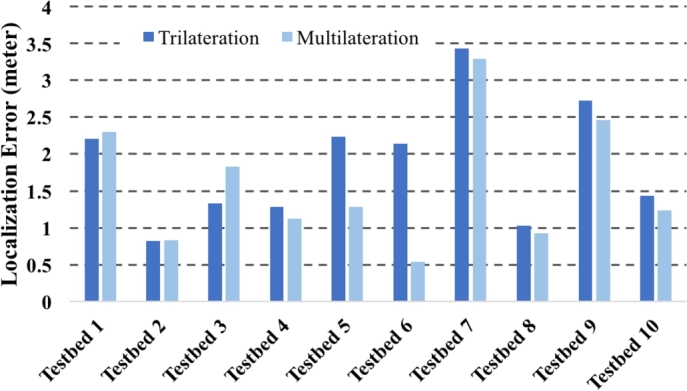
Localization errors in all testbeds for both trilateration and multilateration at presence of one victim.

In VLoc, we position rescuers arbitrarily around a potential search zone with the best intention to put unknown location of the victim within the perimeter of the polygon formed by the rescuers. For a simple analysis in this regard, we consider the presence of just one victim in the search zone. In Fig. 14, Testbed 7 and 9 exhibit higher localization errors compared to others. Although several other factors influence localization error, we are specifically interested here judging the impact of rescuer positioning only. In Testbed 7 and 9, at least one rescuer's position was outdoor. We anticipate that this may have caused higher localization error in these two cases. Note that, in all testbeds, victims are always indoor. What is considered as good positioning of the rescuers? If we consider the victim as the center of a circle, then the problem of positioning rescuers around this victim is similar to the problem of finding any three or four points on the circumference of the circle such that the center is always within the perimeter defined by those points. From Fig. 14, Testbed 5 and 9 have higher localization error. Common to both testbeds is, all rescuers were on the same side of the imaginary diameter through the victim's true position. This happened because the other side was inaccessible.

### 7.4 Impact of increasing number of victims

**Figure 15:**
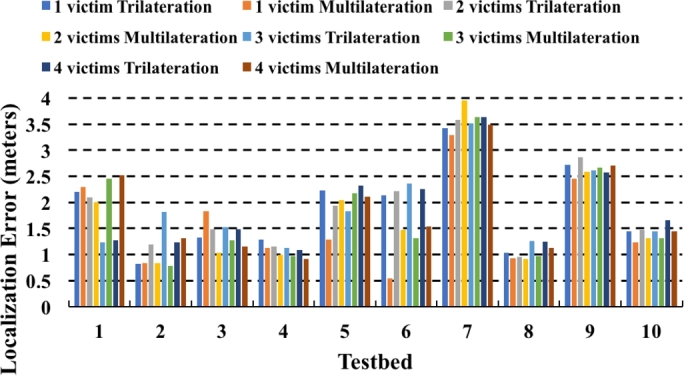
Localization error in all testbeds for both trilateration and multilateration for all victims.

We ran a statistical significance tests to check significance of any factors. We did not find any statistical significance between localization errors in trilateration, multilateration, and response times with increasing number of victims. The relation between increasing number of victims and localization mechanisms (trilateration or multilateration) is not immediately clear from the results in Fig. 15. One could expect to see localization error increasing with increasing number of victims. We anticipate that this is not happening in our case because of lesser number of collisions when APs are beaconing. The Delivery Traffic Indication Message (DTIM3) maintains a 300 ms sleep and 3 ms wakeup cycle to receive APs beacon packet. With DTIM3, it is also possible to consume less power by suspending CPU to save power. Therefore, there are two important things to consider. First, increasing number of victims from four can increase the number of interfering signals. Second, longer DTIM means devices can save their power. Combining both, we notice that longer DTIM will be a desirable choice in our purpose. Most devices (including smartphones and commercial access points) operate in DTIM1 at 100 ms beaconing rate. This may potentially increase interference and hinder VLoc's performance.

### 7.5 Formulating a national policy and database

One of our assumptions is that the rescuers would know the identity of the victims and their phone numbers (sending SMS to switch on hotspot mode). This is not too much to ask, given that most governments and telecommunication companies already store biometric data [1]. In the context of Global South, it is still possible if a national policy is implemented that starts with, for example, industrial workers. A national census on Ready-Made Garment workers in Bangladesh is challenging, however, possible. The outcome of such a census is two-folds. First, it gives rescue teams a number of tentative casualties when hit by an emergency situation. Second, a database created from this census can help others to investigate new design possibilities. A government policy can ask potential smartphone vendors to ship pre-installed app such as VLoc. Although this is unconventional, however, service apps are common that consumers often voluntarily choose to install to avail important services, such as DriveBC app by the British Columbia province in Canada, Noise App in Northern Ireland, VoiceMap HK by Hong Kong government.

## 8 Comparison with other existing approaches

While our solution primarily focuses on the context of global southern countries, its reliability with low-cost components makes it suitable to be applied to any region where the infrastructure and disaster management facilities are not much advanced. Zorn et al. [27] proposed a GSM-based outdoor localization approach that has a location error in the range of 0.1-1.0 m. However, this approach requires additional infrastructure along with pre-calibration. The method proposed by Tassenetto et al. [21] does not require much pre-calibration and infrastructure support, but the localization accuracy is not so good for their case when it comes to victim localization in disastrous sites particularly. Although the model proposed by Giuliano et al. [10] does not require additional infrastructure support, pre-calibration is necessary here. Moreover, the localization accuracy is 1 m which is greater than our proposed approach. RFID based approaches [22] causes much precision loss in localization with nearly 4.137 m. Additional infrastructure and pre-calibration is also necessary.

Our proposed model - VLoc with its Ad hoc and quick pre-calibration, requires no additional infrastructure support. Moreover, the localization error here is only 0.82 m with the incorporation of effective multilateration techniques.

## 9 Limitations and future work

While we acknowledge that emulating disaster-like scenarios such as damaged structures in under-construction buildings and multi-stove kitchen for fire scenarios may not completely inform the design of a real emergency rescue system, it still informs us some basic principles to future designers of such systems. Our choices of testbeds are driven by intentions to emulate disaster-like scenarios “as much as possible”. This investigation of emergency rescue systems for the Global South was rather application centric than informing the theory, since indoor localization is very well studied to date. Consequently, our approach embodies the design of entire rescue mission, not only just victim localization.

In the context of disaster-affected settings, VLoc requires that the phone is not damaged and the phone will still be in victim's possession as long as the rescue mission sustains. While this is a worst possible scenario and potential limitation to any victim localization technique, we believe that out contribution lies in finding a faster way-out for victims of disasters while a communication is still live. This work did not consider the many different ways victims might react in the wake of a disaster, such as running away while his smartphone was on his desk.

During victim probing and identifying potential search zones, rescuers need to “trial” multiple runs of detecting strong Wi-Fi signals. In certain types of disasters such as fire, it may not be possible to continue multiple runs. A possible solution is to adopt multiple probes (more than one) in the same zone, where multiple probes try to identify potential search zones at the same time. If more than one probe agree that a certain zone is the source of strong signal at once, rescuers may move forward to the next steps of rescue process.

The experimental processes are semi-automatic. We collected data in rescuers' devices in a round-robin fashion because of limitations in terms of man power and computing devices. Hence, the true localization time is not known. To overcome this, we plan to explore automating our solution in future.

Additionally, it is worth mentioning that there remains a scope to lessen the response time of our proposed methodology. The response time is calculated through simple summation of time intervals in all steps conducted while locating victims in a search zone. During the process of locating victims in our experimentation, an important part is the rescuers' data collection. In our experimentation, the rescuers' data collection has been done in a round-robin fashion owing to shortage of man power. On the contrary, in real cases, the rescuers' data collection will be done in parallel. Hence, response times in real cases having rescuers' data collection in parallel will be much less than that we have calculated in our experimentation. In addition, we have used locally available low-cost components to construct our system. Using modules with higher computational power will reduce the response time significantly. We plan to explore these aspects in future.

Besides, in our current study, we have focused on only locating victims who are in the same floor. We are yet to investigate the performance of our system when different vertical placements of the victim, e.g., at different floor levels, being underground, etc., are concerned. In the case of having different vertical placements, the impact of ground or floors in signal propagation could exhibit different impacts. Other aspects such as consideration of Fresnel's Zone are also worth exploring in the cases of having different vertical placements. Therefore, in future, we plan to extend our study to locate victims trapped in other floors or in underground. In addition, upon extending to such diversified vertical placements such as multi-floor cases or underground placements, we plan to conduct our experiments in real-life emergency rescue situations.

## 10 Conclusion

In this paper, we took a step to raise a topic of concern to the pervasive computing. The rate at which disasters are happening and the rate at which human lives are lost are both alarmingly high. Despite seeing improvement in indoor localization, very less have been done to improve victim localization in emergency rescue scenarios. The challenges involved in Global South need to be addressed in order to find a suitable solution to this problem. Our study situated upon “how might we” explored the challenges and realistic chances of deploying pre-built infrastructure at disaster sites in the context of Global South. We realized that a solution is necessary that embraces the technology available to the mass in large - smartphones. We conducted a study to better inform the design of an emergency rescue system to leverage smartphones to find location of its owner (victim). We developed a prototype system based on our study and deployed it in several emulated disaster-like scenarios. Experimental results show promising prospects of VLoc in addressing the odds faced by the countries in Global South against frequent disasters.

For future work, a simulation model is needed to simulate post-disaster emergency scenarios before starting a rescue operation. A real-time simulation can provide insights to fire fighters or rescuers and can potentially save lives. Another possible track for future research is a qualitative inquiry that can inform the researchers to further design requirements of an emergency rescue system that can be deployed to a wide population. For example, a qualitative study with Bangladesh Fire Service and Civil Defense can help to inform designing both technology and policy so that this technology can be deployed in real life to save lives.

## Declarations

### Author contribution statement

Taslim Arefin Khan: Conceived and designed the experiments; Performed the experiments; Analyzed and interpreted the data; Wrote the paper.

Tarik Reza Toha: Contributed reagents, materials, analysis tools or data.

Saiful Islam Salim: Contributed reagents, materials, analysis tools or data; Wrote the paper.

Md Toki Tahmid: Analyzed and interpreted the data; Wrote the paper.

A. B. M. Alim Al Islam: Conceived and designed the experiments; Analyzed and interpreted the data.

### Funding statement

This research did not receive any specific grant from funding agencies in the public, commercial, or not-for-profit sectors.

### Data availability statement

Data included in article/supplementary material/referenced in article.

### Declaration of interests statement

The authors declare no conflict of interest.

### Additional information

No additional information is available for this paper.
